# Association of gastrointestinal microbiome and obesity with gestational diabetes mellitus-an updated globally based review of the high-quality literatures

**DOI:** 10.1038/s41387-024-00291-5

**Published:** 2024-05-21

**Authors:** Jiahui Li, Min Wang, Shuai Ma, Zhong Jin, Haonan Yin, Shuli Yang

**Affiliations:** Department of Gynecology and Obstetrics, The Second Hospital of Jilin University, Changchun, 130000 Jilin China

**Keywords:** Gestational diabetes, Bacteria

## Abstract

**Objectives:**

The purpose of this review is to investigate the relationship between gastrointestinal microbiome, obesity, and gestational diabetes mellitus (GDM) in an objective manner.

**Methods:**

We conducted a thorough and comprehensive search of the English language literatures published in PubMed, Web of Science, and the Cochrane Library from the establishment of the library until 12 December 2023. Our search strategy included both keywords and free words searches, and we strictly applied inclusion and exclusion criteria. Meta-analyses and systematic reviews were prepared.

**Results:**

Six high-quality literature sources were identified for meta-analysis. However, after detailed study and analysis, a certain degree of heterogeneity was found, and the credibility of the combined analysis results was limited. Therefore, descriptive analyses were conducted. The dysbiosis of intestinal microbiome, specifically the ratio of Firmicutes/Bacteroides, is a significant factor in the development of metabolic diseases such as obesity and gestational diabetes. Patients with intestinal dysbiosis and obesity are at a higher risk of developing GDM.

**Conclusions:**

During pregnancy, gastrointestinal microbiome disorders and obesity may contribute to the development of GDM, with all three factors influencing each other. This finding could aid in the diagnosis and management of patients with GDM through further research on their gastrointestinal microbiome.

## Introduction

Trillions of microbial cells essential to human health are present in the human body. The gastrointestinal microbiome, located on the surface of the intestinal mucosa, is the largest microbial ecosystem in the human body and is involved in epithelial homeostasis, energy harvesting and immune development [1, 2], and has a major impact on nutrient and energy metabolism, with significant implications for human health [3]. Based on their relationship to host health, the gastrointestinal microbiome can be categorized into beneficial, harmful and conditionally pathogenic bacteria [4]. There is no single optimal gastrointestinal microbiome composition, as it varies from individual to individual, and it is important to maintain a healthy balance between the host and the pathogens in order to achieve optimal metabolic and immune functions [5]. Changes or ecological disturbances in the normal composition of the gastrointestinal microbiome are defined as an imbalance between commensal and pathogenic bacteria [6], and the gastrointestinal microbiome can regulate each other to maintain the abundance and diversity of the microbiome in a dynamic equilibrium [4]. Due to changes in the body’s endocrine status, immune function, age profile, dietary structure, environmental and genetic factors, the gastrointestinal microbiome can become dysbiotic and show changes in abundance and diversity, this can lead to the development of metabolic disorders such as obesity and diabetes [6].

Overweight and obesity are significant global health issues and are significant risk factors for GDM [7]. Overweight and obesity have a strong association with adverse pregnancy outcomes. An increase in pre-pregnancy body mass index (BMI) significantly increases the risk of fetal miscarriage, neonatal hypoglycemia, fetal malformations, large for gestational age, macrosomia, and cesarean section [8, 9]. Obesity reduces the number of insulin receptors and causes receptor defects, leading to insulin resistance (IR) and higher fasting insulin levels. This affects glucose transport, utilization, and protein synthesis. Women are particularly susceptible to GDM due to excessive nutritional intake during pregnancy.

GDM is one of the most common complications of pregnancy and is defined as the first occurrence of diabetes during pregnancy due to abnormal glucose metabolism [7, 10]. The International Diabetes Federation estimates that the global prevalence of diabetes will increase to 10.2 percent (578 million people) by 2030 and to 10.9 percent (700 million people) by 2045 [11]. In normal pregnancy, especially towards the third trimester of pregnancy(T3), beta(β) cells secrete more insulin to compensate and maintain normal blood glucose levels due to IR. However, in GDM, certain changes lead to a reduction in insulin sensitivity, impairment of insulin secretion and the development of carbohydrate intolerance [12]. The molecular mechanisms by which these changes take place are still not clear.

In recent years, research into the relationship among the gastrointestinal microbiome and obesity and GDM has become a major topic in medicine, but the results of these studies on the upregulation or downregulation of the gastrointestinal microbiome are not consistent because the subjects were from different countries and races, and the tests used were different. Due to the high degree of heterogeneity of the cohort studies in these areas, it was not possible to perform combined analyses. Therefore, this review aims to provide a comprehensive overview of these three areas of research, to further our understanding of GDM, to identify new targets for treatment of GDM, and ultimately to provide guidelines for clinicians.

## Composition and characteristics of the gastrointestinal microbiome during pregnancy

The microbiome, which is the collection of all gastrointestinal microbial genes in an individual, is an order of magnitude larger than the human genome [13, 14]. The adult intestinal is colonized by at least 1800 genera and approximately 15–36,000 species of bacteria [15]. The human gastrointestinal microbiome consists of four main groups: Firmicutes, Bacteroides, Actinomycetes, and Proteus [16]. The ratio of Firmicutes to Bacteroides(F/B) is a crucial parameter that reflects dysbiosis of the gastrointestinal microbiome [17]. According to molecular analyses targeting 16S rRNA, the fecal samples of healthy human volunteers contained bacteria primarily from two phyla: Firmicutes and Bacteroidetes. These bacteria were predominantly anaerobic species [18]. Compositionally, the gastrointestinal microbiome of the entire pregnant population is also dominated by members of Firmicutes and Bacteroidetes, which account for approximately 90% of the microbial environment in the intestine [5, 19]. Currently, there is a great deal of variation in the study of gastrointestinal microbiome in various countries, possibly due to factors such as study populations from different races, different ages, the use of different test methods, contamination of study reagents, and statistical differences.

## The role of bile acids

**Fig. 1:**
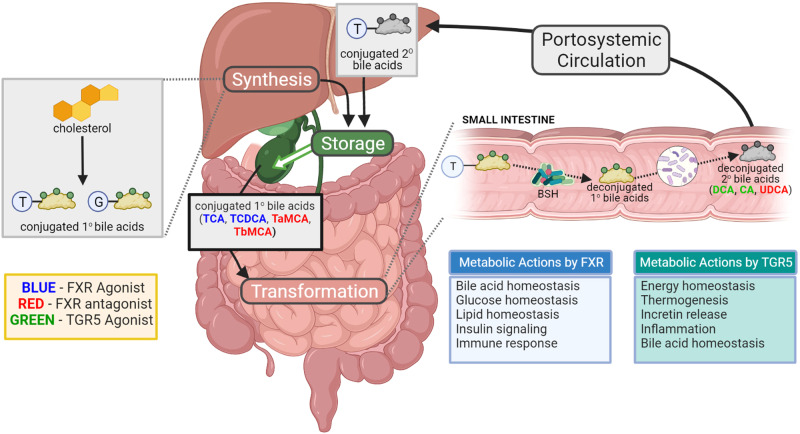
Bacterial BA biotransformation [21]. Cholesterol is converted to primary BAs in the liver. Primary BAs are conjugated with primarily taurine in mice or glycine in humans before being transported to the gallbladder for storage in the form of bile. On ingestion of dietary fats, primary conjugated BAs (within bile) are released into the gut lumen to aid lipid absorption. Bacteria with BSH deconjugate BAs, thereby weakening their soap-like qualities. This allows other microbiome members to further modify them into secondary BAs. Some secondary BAs can be transported back to the liver, where they are then conjugated. The interaction between the gut microbiome and BAs leads to modulation of FXR and TGR5 agonists and antagonists, and thus, allows the gut microbiome to affect host metabolism (Note: T taurine, G glycine. In humans: TCA taurocholic acid, TCDCA taurochenodeoxycholic acid. In mice: TαMCA tauro-α-uricholic acid, FXR farnesoid X receptor, TGR5 G protein-coupled BA receptor 1).

It is worth noting that bile acids (BA), which are a type of amphiphilic steroid produced in the liver and modified by the microbiome, are increasingly recognized as contributing to the pathogenesis and progression of metabolic diseases such as obesity and diabetes. BAs promote the absorption of intestinal fat. They also have hormone-like functions by activating nuclear and membrane-bound receptors, which regulate glucose, lipid and energy metabolism, intestinal integrity and immunity [20]. The BA biotransformation process is shown in Fig. 1 [21].

Glucose homeostasis is a crucial physiological process affected by BAs. BA signaling pathways, such as the nuclear hormone receptor FXR [22] and TGR5 [23], are highly conserved metabolic regulatory pathways between mouse models and humans, and they affect IR. Additionally, BA regulates dozens of genes involved in metabolic homeostasis through the activation of fibroblast growth factor 19 in humans [24]. Studies in mice have shown that FXR regulates the release of glucagon-like peptide-1 (GLP-1) [25], This peptide promotes gluconeogenesis [26] and browning of white adipose tissue in the liver and muscle [27]. The influence of diet on BA signaling is also evident. Studies on FXR in various animal models of metabolic disorders have yielded inconsistent results. However, several studies have demonstrated that mice lacking FXR on a regular diet develop hyperglycemia and hypercholesterolemia [28]. Additionally, feeding FXR-deficient mice a high-fat diet prevented obesity and improved glucose homeostasis [29, 30]. TGR5 is expressed in various organs including the gallbladder, lung, spleen, liver, bone marrow, and placenta [28]. It is also present in intestinal L cells, immune cells such as Kupffer cells, muscle, and brown adipose tissue [21]. TGR5 activation in brown adipose tissue promotes calorie production from stored fat. In L cells, TGR5 activation affects glucose homeostasis primarily through the secretion of GLP-1 [21].

Circadian rhythms also affect human glucose metabolism, with a 34% decrease in insulin sensitivity at night compared to the morning [31]. Disturbances in circadian rhythms are considered a significant factor in metabolic disorders. The roles of intestinal microbiome and BA metabolism have received significant attention. A recent study highlights the importance of maintaining proper oscillations of Ursodeoxycholic acid in a lean state to synchronize insulin sensitivity oscillations [32]. However, the function of the gut microbiota-bile acid axis in regulating circadian rhythms of metabolic homeostasis remains largely unknown and requires further exploration in the future.

## The relationship among gastrointestinal microbiome, obesity and GDM

During pregnancy, around 20% of patients develop pre-diabetes or type 2 diabetes Mellitus [33]. Hormone levels undergo various changes during pregnancy, particularly with a significant increase in luteinizing hormone and estrogen levels, which produce many physiological effects. These hormone levels may affect the composition of the microbiome [34]. In addition, changes in modern lifestyles and the use of antimicrobial drugs have led to a decrease in the diversity of the gastrointestinal microbiome in many populations in developed countries [35]. Dysbiosis of the gastrointestinal microbiome can have several adverse effects on the organism, such as imbalances between Firmicutes and Bacteroidetes, which can lead to obesity and diabetes [36].

This review follows the Preferred Reporting Items for Systematic Reviews and Meta-Analyses protocol. We conducted a comprehensive search for both published and unpublished literature in English up to 12 December 2023, using databases such as PubMed, Web of Science, and the Cochrane Library. In order to gather the literature, we established rigorous inclusion and exclusion criteria and employed a search strategy that combined keywords with free words or synonyms.

Inclusion criteria

1. The study compared women during pregnancy with GDM (experimental group) to those with normal glucose tolerance (control group), while also examining differences between women with normal weight and those who were overweight or obese.
2. Fresh feces were collected from the subjects in the early morning, and fasting venous blood was drawn to analyze gastrointestinal microbiome and measure BMI.
3. The study included both experimental and control groups to investigate indicators of gastrointestinal microbiome, levels of obesity, and blood glucose levels.
4. Make sure that experimental and control groups were comparable.
5. Use randomized controlled trials, case-control studies, or cohort studies.
6. The literatures included in this study were written in English.

Exclusion criteria

1. Studies on women with multifetal pregnancies or less than 10 cases.
2. Studies for which the full text was not available or from which no valid data could be extracted.
3. This section excludes studies in the form of case studies, reviews, and lectures.

Considering that this is the first systematic review so far to study the association of gastrointestinal microbiome and obesity with GDM, this direction was registered with PROSPERO (registration number: CRD42023486272). After conducted a thorough literature search and analysis (see Table 1 for the search process), a total of 6 relevant documents were identified for this study (see Table 2 for the basic characteristics of the included documents), and there was less comparability and more heterogeneity between the groups, which made it impossible to perform a combined analysis. Considering the above, a descriptive analysis of these few high-quality documents is presented below.

**Table 1:**
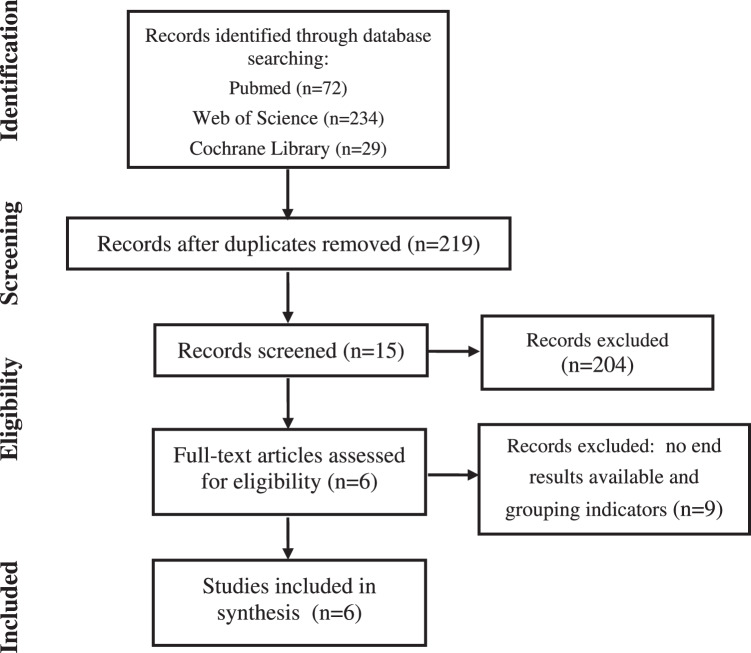
Process of literature search.

**Table 2:** Basic characteristics of the included literature.

First author	Source of population	Type of literature	Sample size and grouping
Yao Su [36]	Shanghai, China	Randomized controlled trials	*N* = 122 (consisting of 71 normal pregnant women, 27 with GDM alone, 17 with hyperrecombination alone, and 7 with both GDM and hyperrecombination)
Zhi-ying Song [4]	Shanxi, China	Randomized controlled trials	*N* = 79 (consisting of 42 normal pregnant women, 12 with GDM alone, 16 with super-reconstitution alone, and 9 with GDM and super-reconstitution)
Thomas [39]	Australia	Randomized controlled trials	*N* = 58 (GDM group, *n* = 29; Non-GDM group, *n* = 29)
Bahiyah [40]	Malaysia	Prospective observational study	*N* = 38 (GDM group *n* = 12, Non-GDM group *n* = 26. Further groupings: Underweight, Normal BMI, Pre-obese, Obese)
Patricia [42]	Sao Paulo, Brazil	Randomized controlled trials	*N* = 115 (GDM group *n* = 56, Control group *n* = 59) (all obese or overweight)
Marketa [41]	Olomouc, Moravia	Randomized controlled trials	*N* = 104 (consisting of a normal pregnant group, *n* = 22; GDM1 group, *n* = 29; GDM2 group, *n* = 31; GDM3 group, *n* = 22)

It has been found that most studies on the involvement of gastrointestinal microbiome in metabolic diseases rely on the use of 16S rRNA marker genes [37]. The 16S rRNA genes of bacteria are made up of conserved and variable parts, the latter being specific to a given strain. Sequences from the V3 to V4 parts of the 16S rRNA gene are generally accepted as representative of all 16S rRNA sequences, and the analysis of sequences from the V3 to V4 parts of the 16S rRNA gene is used to determine the taxonomy of each bacterial species [38]. Only one of the above six papers [39] sequenced the V6–V8 variable region of 16S rRNA, but ultimately it was the 16S rRNA that was investigated as a means to speculate on its interaction with host metabolism.

Two studies have suggested a correlation between the composition of gastrointestinal microbiome and both GDM and weight status [36, 40]. Based on different periods of pregnancy, Zhi-ying Song [4] concluded that significant differences in the species composition of the gastrointestinal microbiome existed between the purely overweight group and the group of normal women in T3. In a study of a group of people who were both obese or overweight, Marketa [41] concluded that significant differences in the gastrointestinal bacterial microbiota of different GDM pathogenesis groups existed in the first trimester of pregnancy. The two remaining studies [39, 42] comparing the composition of gastrointestinal microbiome between GDM and women in pregnancy without GDM (non-GDM) groups found no significant differences. In their analysis of the intestinal microbiome and weight, Yao Su and colleagues [36] discovered that the ratio of F/B in the ultra-restructured gastrointestinal microbiome of GDM was approximately 3:5, which is the opposite of other groups. Zhi-ying Song [4] found a negative correlation between pre-pregnancy BMI and Lactobacillus, as well as between weight gain during pregnancy and Desulfovibrio in Proteobacteria. (Table 3 presents the specific differences in the indicators of gastrointestinal microbiome between the groups.)

**Table 3:** Differences in indicators of intestinal microbiome between groups.

First author	Global differences in indicators of intestinal microbiome	
Yao Su [36]	Four groups of genus-level biomarkers of intestinal microbiome	
	Normal pregnant women group	Clostridia and Clostridiales
	GDM alone group	Ruminococcaceae_UCG014
	Ultra-recombinant alone	Rahnella
	GDM ultra-recombined	Deinococcus, Obscuribacteralesjun, Clostridium_sensu_stricto_3, and Terrisporobacter
Zhi-ying Song [4]	Characteristic intestinal microbiome at 24 weeks of gestation	
	Normal pregnant women group	g-Ruminococcus
	GDM alone group	o_Desulfovibrionales, f_Desulfovibrionaceae, c_ Deltaproteobacteria, and g_Anaerostipes
	GDM alone	p_Verrucomicrobia, c_Verrucomicrobiae, o_Verrucomicrobiales, f_Verrucomicrobiaceaem and g_Akkermansia
	GDM ultra-recombined	g_Lactobacillus
	Characteristic intestinal microbiome at 37 weeks of gestation	
	Ultra-recombined alone	f_Enterobacteriaceae and o_Enterobacteriales;
	GDM ultra-recombined	g_Eubacterium, g_Pyramidobacter and f_Dethiosulfovibrionaceae.
Thomas [39]	GDM group (at 28 weeks’ gestation)	The g_Blautia in the f_Lachnospiraceae↑
		The p_Bacteroidales ↓ , Lachnospiraceae↓
		The g_Eggerthella was uniquely associated in women with GDM (abundance: 0.126; occurrence: 41%).
		The genera Unclassified.RF39, Desulfovibrio and Dehalobacterium were uniquely associated in euglycaemic women.
Bahiyah [40]	GDM group	Genera Acidaminococcus ↑ , Clostridium ↑ , Megasphaera ↑ , Allisonella↑
		Barnesiella ↓ , Blautia↓
	Obese patients	Megamonas ↑ , Succinatimonas ↑ , Dialister↑
	Normal and underweight patients	Clostridia (Papillibacter, Oscillibacter, Oscillospira, Blautia, Dorea) ↑, Bacteroidia (Alistipes, Prevotella, Paraprevotella)↑
Patricia [42]	GDM group	g_Bacteroides↑
	Third trimester of pregnancy	Bifidobacterium ↑ , Peptococcus↑
Marketa [41]	Normal blood glucose	f_Prevotellaceae ↑ , order Fusobacteriales ↑ , g_Sutterella↑
	Abnormal FGP	genera Enterococcus↑
	Abnormal OGTT	Erysipelotrichaceae UCG-003↑

Note: the genus: g_; the order: o_; the family: f_; the class: c_; the phylum: p_.

The diagnosis of GDM is primarily based on a glucose tolerance test. This involves measuring fasting glucose levels, as well as glucose levels at 1 h (OGTT_1h) and 2 h (OGTT_2h) after consuming a glucose solution. (Criteria: At least one of the three blood glucose values must be equal to or greater than 5.1 mmol/L, 10.0 mmol/L, and 8.5 mmol/L, respectively.) The test is typically performed between 24 and 28 weeks of gestation and follows the guidelines developed by the International Association of Diabetes and Pregnancy Research Groups [43]. The correlation analyses conducted by Zhi-ying Song and others [4] found that the composition and abundance of gastrointestinal microbiome have an impact on GDM. Specifically, blood glucose values at OGTT_1 h and OGTT_2 h were positively correlated with Bacteroides, and negatively correlated with Prevotella. It is important to note that these findings are based on objective data and do not include any subjective evaluations. The study In Patricia [42] found a positive correlation between the abundance of Christensenellaceae and Enterobacteriaceae with plasma glucose levels one hour after OGTT. Conversely, Enterococci were negatively correlated with plasma glucose levels two hours after OGTT. These findings suggest potential implications for the diagnosis and treatment of GDM.

A cohort study by Dualib et al. [42] concluded that in relation to the development of GDM, alpha(α) and β diversity did not differ between the GDM and Non-GDM groups, despite differences in the relative abundance of specific bacteria. In our study, Thomas et al. [39] found that no significant changes in the relative abundance of major bacterial taxa were detected between women in health and women with GDM at 28 weeks’ gestation, and that the occurrence of GDM was associated with a decrease in Shannon diversity (*p* = 0.02) but no different clustering as measured by β-diversity, and that at 28 weeks’ gestation the women with GDM had a decreased microbial richness and evenness.

In Patricia’s study [42], the abundance of bifidobacteria and peptidococci increased in the third trimester of pregnancy, but there was no difference in α-diversity or overall microbiota structure between the two groups. These results suggested a degree of inconsistency, which could be due to differences in the subject populations, experimental error, or the comparability of the groups. Given that Patricia [42] included women who were overweight or obese regardless of when GDM was diagnosed by abnormal blood glucose, it is possible that the difference in BMI contributed to the increased gastrointestinal microbiome abundance, but this is only a preliminary hypothesis and further studies with larger sample sizes are needed to support this conclusion.

Research has indicated that individuals with a low abundance of gastrointestinal microbiome are more susceptible to dyslipidemia [44]. In this study, Thomas and colleagues [39] found that the GDM group had elevated levels of VLDL, triglycerides, venous glucose, HOMA-IR, and C-peptide at 28 weeks of gestation when compared to the control group. In a randomized controlled trial, Marketa [41] also found that Escherichia/Shigella had a positive correlation with plasma lipid levels, while Subdoligranulum had a negative correlation with plasma lipid levels in the GDM group. Coprococcus, Akkermansia, Methanobrevibacter, Phascolarctobacterium, and Alistipes were found to have a positive correlation with acetate, valerate, 2-hydroxybutyrate, and 2-methylbutyrate levels, respectively. In summary, these lipid metabolism abnormalities contribute to the development of obesity and GDM, but the specific indicators may vary among individuals.

**Fig. 2:**
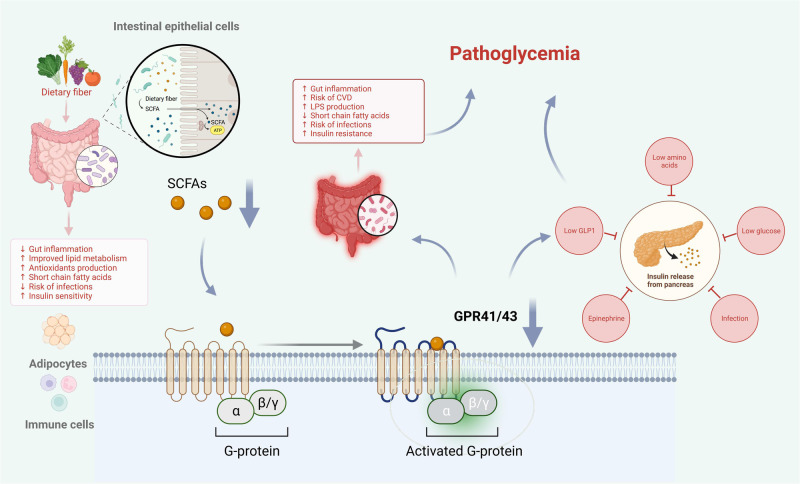
Lipid molecules regulate host metabolism through GPR41 and GPR43 receptor linkage.

Short-chain fatty acids (SCFAs) play a crucial role in glucose homeostasis by providing additional energy from undigested food. Butyrate, acetate, and propionate are the three main SCFAs produced by gastrointestinal microbes during the fermentation of nondigestible dietary fiber in the large intestine. SCFAs are most concentrated in the cecum and proximal colon, with concentrations decreasing towards the distal colon [21]. The type of SCFAs produced and the diet determine the metabolic pathways triggered through various receptors. SCFAs influence the regulation of host lipid and glucose metabolism through G protein-coupled receptors (GPCRs) linkages, such as GPR41 and GPR43 [45], as shown in Fig. 2. Disturbances in gastrointestinal microbiome can lead to a reduced intestinal anti-inflammatory response. Low levels of SCFAs can also reduce the activation of GPCRs, leading to reduced activation of GPR41 and GPR43, which can generate intestinal inflammation, insulin resistance, and ultimately, diabetes [46]. Studies have shown that GPR43-deficient mice become obese even on a normal diet, while mice that specifically overexpress this receptor in adipose tissue remain lean [47]. Additionally, GPR43 activation promotes the secretion of GLP-1 in the intestine, enhancing insulin sensitivity [48]. SCFAs deficiency can cause a loss of tight junctions and increased enterocyte permeability. This can lead to increased absorption of bacterial endotoxins, such as lipopolysaccharide, which in turn can cause the production of pro-inflammatory cytokines. These factors can predispose women to IR and GDM [49].

Normal gastrointestinal microbiome has a positive impact on host metabolism. SCFAs activate GPCRs like GPR41 and GPR43. These receptors are expressed in various cell types, including intestinal epithelial cells, adipocytes, and immune cells [33]. Disruptions in the gastrointestinal microbiota can weaken the intestinal anti-inflammatory response. Low levels of SCFAs can also decrease GPCR activation, potentially resulting in intestinal inflammation, IR, and ultimately GDM.

## Relationship between intestinal microbiome and GDM

Microbial abundance in women with GDM compared to non-GDM is either reduced [50–52], unchanged, or elevated [19]. However, there is currently no specific microbiota that can predict the development of GDM. Generally, pregnancy leads to increased bacterial loads and significant alterations in the composition of the intestinal microbiome [53]. The composition of intestinal microbiome during the first trimester of pregnancy(T1) is similar to that of women in health and nonpregnancy [53]. Microbiome disorders are highly characteristic in patients with GDM, particularly in mid-pregnancy(T2), and can be used as a predictor of GDM [54]. Significant alterations in the composition of the intestinal microbiome were observed in women during pregnancy compared to non-pregnant women, and from T1 to T3 [55, 56]. The late pregnancy intestinal microbiome has been found to cause weight gain, insulin resistance, and a greater inflammatory response when transferred to germ-free mice compared to the early pregnancy microbiota [55]. In T3, Koren et al. found an increase in the abundance of the Actinobacteria and Aspergillus phyla, and a decrease in the Faecalibacterium [55]. Ferrocino et al. [54] discovered that individuals with GDM had an increased abundance of Blautia, Butyricicoccus, and Clostridium, as well as a decreased abundance of Bacteroides, Collinsella, and Rikenellaceae during T2 compared to T3 [19].

However, the gastrointestinal microbiome of patients with GDM may be abnormal at several levels, including the phylum and genus levels. According to a study [19], the GDM cohort had a higher abundance of Actinobacteria at the phylum level and Collinsella, Roseburia and Desulfovibrio at the genus level. According to Koren [55], significant changes are identified by a decrease in individual richness (α-diversity), an increase in intersubject diversity (β-diversity), and altered abundance of certain species.

An operational taxonomic unit (OTU) is a set of uniform markers used to represent taxonomic units, such as phylum, order, family, genus, and species. OTUs are created for the purpose of facilitating analysis in phylogenetic or population genetics studies [26, 57]. In a comparative study of intestinal microbiome differences between healthy pregnant women and GDM patients, two studies analyzed 18 [58], and 17 [19] bacterial OUTs, respectively. The major differences in dysbiosis OUTs were attributed to the Firmicutes (72.2% and 88.2%). This suggests that alterations in the Firmicutes are a characteristic hallmark of GDM. Therefore, strains specific to the Firmicutes require urgent exploration in the future.

In a comparative study between women during pregnany in a normal state and those with diabetes, Xing et al. found that GDM subjects had a higher abundance of Lachnospiraceae family OTUs and a lower abundance of Enterobacteriaceae and Rumatococcaceae. Two Lachnospiraceae OTUs (247 and 672) were positively correlated with OGTT_1h at 24–28 weeks of gestation, and bacterial OTUs (e.g., Enterobacteriaceae_OTU 123 and Rumatococcaceae_OTU 93) were associated with FBG levels at 12 weeks of gestation [58]. Intestinal Lachnospiraceae bacteria have been suggested to be positively associated with type 2 diabetes Mellitus [59]. A study conducted in China found that, at the species level, the relative abundance of Clostridium_spiroforme, Eubacterium_dolichum, and Ruminococcus_gnavus was positively correlated with FBG, while Pyramidobacter_piscolens was negatively correlated with FBG [57]. A previous animal study also reported a positive correlation between Lachnospiraceae OTUs and blood glucose levels [60].

Ruminococcus gnavus is an anaerobic bacterium that is Gram-positive. It belongs to the Firmicutes, it is also a member of the family Lachnospiraceae. An increasing number of enteric and extra-enteric diseases are associated with this bacterium [61]. Zhi-ying Song [4] and Yao Su [36] found that g-Ruminococcus was a characteristic biomarker for the normal pregnant women group and Ruminococcaceae_UCG014 was a characteristic biomarker for the GDM-only group, respectively. The study suggests that Ruminococcaceae play a role in energy metabolism, insulin signaling, and inflammatory processes. It also found that an increase in the relative abundance of Ruminococcaceae is associated with higher FBG concentrations and TR, which increases the risk of developing GDM [62]. These findings indicate the potential predictive value of specific microbial combinations for GDM.

There is no predictive value of blood glucose values for the development of severe GDM. Furthermore, differences in the severity of GDM correspond to changes in gastrointestinal microbiome [63]. In a prospective longitudinal study conducted in Chiang Mai, Thailand, there were no differences in FBG, OGTT_2h, and glycated hemoglobin levels at diagnosis between patients with diet-controlled GDM and those requiring insulin therapy [63]. However, unlike the literature included in this study, GDM patients who ultimately required insulin therapy had higher levels of Clostridium difficile [63]. Lactobacillus has long been considered beneficial to the host by attenuating intestinal mucosal barrier dysfunction, remodeling intestinal microbiota composition, and reducing systemic inflammation [64–66]. However, there are conflicting accounts of its effects on GDM patients.

This study concludes that g_Lactobacillus may be a characteristic gastrointestinal microbiome of overweight GDM patients, distinguishing them from other patient groups [4]. A cross-sectional study found that GDM patients had lower levels of Lactobacillus casei than non-GDM controls before delivery [67]. Another study showed that the relative abundance of specific Lactobacillus at diagnosis was higher in women with GDM than non-GDM [68]. The varying results regarding different bacilli and blood glucose levels can be attributed to different subgroups of bacilli sequences. Therefore, it is important to explore the microbiome further in order to gain a better understanding of the role of various bacilli in female patients with GDM.

## The relationship between gastrointestinal microbiome and obesity

Animal and human studies have shown that obesity is associated with an imbalance or ecological dysbiosis of the intestinal microbiome [69], as the imbalance between energy consumption and depletion favors the prevalence of disease-causing bacteria [70], but the role of the intestinal microbiome in the development of this disease and whether there is a causal relationship remains controversial [37]. The gastrointestinal microbiome plays a crucial role in the absorption of nutrients, in digestion and metabolic activities, as well as in the efficiency and storage of energy [71]. However, changes in the composition of various factors caused by the microbiota (ecological dysbiosis) may have adverse long-term effects, leading to diseases such as obesity, intestinal inflammation, diabetes and metabolic syndrome in the host organism and in future generations [72]. The effect of the intestinal microbiota on host metabolism was first demonstrated in a 2004 study of germ-free mice, which found that conventionally raised mice had more total body fat than those raised in germ-free conditions [73]. Studies evaluating the increased ratio of F/B following microbiota transplantation in obese individuals have not yet produced consistent results [74, 75].

The maternal intestinal microbiota composition changes during pregnancy and breastfeeding due to maternal metabolism adjustments caused by the increased demands of the developing fetus and postnatal infant, as well as the mother’s own physiological changes [76]. Dysbiosis of the intestinal microbiome is prevalent in obesity and is characterized by a reduction in the diversity [44] and abundance of the intestinal microbiome in obese individuals [77]. Studies have shown that individuals with low intestinal microbiome abundance are more susceptible to obesity, IR [78], and dyslipidaemia [44]. In patients with obesity, the levels of mucinophilic Akkermansia muciniphila, Faecalibacterium prausnitzii, and Bacteroides were found to be decreased [79–81], while the abundance of fungal phyla was significantly increased [82–84].

A follow-up study was conducted in Finland with 256 women. The study found that overweight and obese mothers had a higher relative abundance of the Firmicutes, and there was a trend towards a higher ratio of F/B [85]. Additionally, the ratio of F/B decreased after weight loss in obese individuals [83]. It has been suggested that the higher relative abundance of Prevotella detected in women during pregnancy with obesity, compared to those who are overweight, may contribute to glucose metabolism through the metabolites produced [86].

Akkermansia is a Gram-negative, anaerobic, elliptical bacterium that degrades mucin and inhabits the outer mucus layer of the intestinal barrier [37, 87]. The mechanisms by which mucin regulates obesity and glucose levels have not been fully elucidated. In humans, its abundance and genetic richness are positively correlated with healthy metabolic states, including better body fat distribution and absence of metabolic syndrome [88, 89]. A previous study demonstrated that Akkermansia enhances thermogenesis and GLP-1 secretion, while reducing the expression of proteins involved in adipocyte differentiation. Additionally, it decreases the gene expression of glucose and fructose transporter proteins in the jejunum, indicating a reduction in carbohydrate absorption [90–92]. In conclusion, while most studies suggest a beneficial role for Akkermansia in metabolic profiling, its effects may be dual depending on dietary patterns [37]. Moran noted that consistent observations in the human intestinal microbiota and its interactions with diet and genetics suggest that the microbial diversity of individuals with a high BMI or obese individuals is lower. According to the study, Christensenellaceae, Oscillospira, and Rikenellaceae were more prevalent in individuals with a normal body weight, while Bifidobacteria and Akkermansia were less abundant in those with altered metabolism [37].

## Relationship between obesity and GDM

Maternal adiposity increases significantly during pregnancy due to the increased nutritional needs of the fetus and the demands of the mother’s own metabolism. This can lead to GDM due to the development of IR. Obesity can also affect GDM through other mechanisms, including impaired β-cell function and chronic low-grade inflammation [93, 94]. This inflammation is mainly manifested as dyslipidemia and a pro-inflammatory state during pregnancy [95, 96]. Prospective studies have linked a range of fatty acids, phospholipids, lipoproteins, certain glycolipids, and cholesterol with incident GDM [97]. In a recent study of 1008 women’s lipidomic, it was observed that seven out of ten lipids associated with BMI (four LPCs, two TGs, and one SM) were linked to the risk of GDM, even after adjusting for maternal BMI [98]. Additionally, gestational weight gain has been experimentally confirmed to be associated with an increased risk of developing GDM [99]. A Danish study found that 11 OTUs were associated with gestational weight gain, with the majority being Clostridiales (7 of 11 OTUs), when diabetic status was not taken into account. Lower weight gain was associated with 7 OTUs, including a Christensenella OTU (OTU_63) and an Alistipes OTU (OTU_128). Weight gain was associated with 4 OTUs, including an Eisenbergiella OTU (OTU_258) and a Lactobacillus OTU (OTU_80) [19].

Stored upper body fat in pregnant women with obesity can increase the concentration of free fatty acids and lipotoxicity. This can lead to inflammation, endothelial dysfunction, and reduced trophoblastic invasion, ultimately decreasing placental metabolism and function [100]. The placenta serves as the sole interface between the mother and the fetus, making it a crucial organ for the exchange of gases and nutrients between the two. Specific changes occur in the structure of the placenta in pregnant women who are obese and diabetic, including increased weight, angiogenesis, and slower chorionic villus maturation. These structural abnormalities lead to functional abnormalities, which worsen metabolic abnormalities during pregnancy [101]. Abnormal protein expression in the placenta can cause insulin antagonism, resulting in abnormal insulin resistance and glucose metabolism [102]. Five proteins, namely very low density lipoprotein receptor, aquaporin-1, platelet factor 4, peptidyl prolyl isomerase, and malonyl cofactor-acyl carrier protein transacylase, have been associated with altered placental function, placental vascular dysfunction, and placental inflammation and its complications in patients with GDM [103–105]. Reduced very low density lipoprotein receptor levels may promote GDM by inhibiting the placenta’s ability to remove cholesterol [103]. Maternal IR is a pathophysiological condition that causes changes in the growth and efficiency of the placenta in pregnant women, especially those who are obese and diabetic [106]. IR may enhance chorionic cell proliferation and increase placental size, but expansion of immature chorionic villi may reduce the efficiency of placental transport mechanisms, leading to placental insufficiency [107, 108]. Furthermore, during pregnancy, the placenta secretes pregnancy-specific hormones such as human chorionic gonadotropin, human placental lactogen, and human placental growth hormone, as well as increased levels of prolactin, estradiol, and cortisol into the maternal circulation. These hormones can affect glucose metabolism and lead to the development of diabetes. It is important to note that this is a complex process and further research is needed to fully understand the mechanisms involved. The rapid recovery of glucose homeostasis immediately after placenta expulsion at delivery demonstrates the significant role of the placenta in GDM with obese patients.

Recent data suggest that exosomes, which are membrane-derived nanovesicles, may play a role throughout pregnancy. This includes mediating placental responses to hyperglycemia and insulin sensitivity. Patients with GDM have been found to have higher levels of circulating exosomes, both overall and of placental origin, during gestation compared to normal pregnancies [109]. Additionally, hyperglycemia has been shown to increase the release of exosomes from trophoblast cells in early pregnancy [110], indicating a correlation between maternal metabolic status during pregnancy and circulating levels of placental exosomes.

It has also been suggested that hyperactivation of adipose tissue plays an important role in the pathogenesis of GDM. Lipocalin is a protein produced in large quantities by adipose tissue. It enhances insulin sensitivity, exerts anti-inflammatory effects, and reduces plasma glucose levels [111]. Lipocalin is thought to play a pivotal role in the regulation of systemic glucose homeostasis [112]. Lipocalins are expressed and synthesized primarily in maternal adipose tissue, but not via the placenta, and do not enter the fetal circulation [113]. Deletion of the lipocalin gene leads to impaired insulin tolerance [114]. Previous studies have shown that low levels of lipocalin in pregnant women are associated with reduced maternal insulin sensitivity during pregnancy [115]. These findings suggest that lipocalin may play a role in insulin sensitivity during pregnancy. In studies conducted in various populations, including South India [116] and Iran [117] maternal serum lipocalin levels were significantly lower in patients with GDM. The use of lipocalin as a prognostic biomarker for GDM risk is currently a topic of debate due to inconsistent results.

## Conclusion

**Fig. 3:**
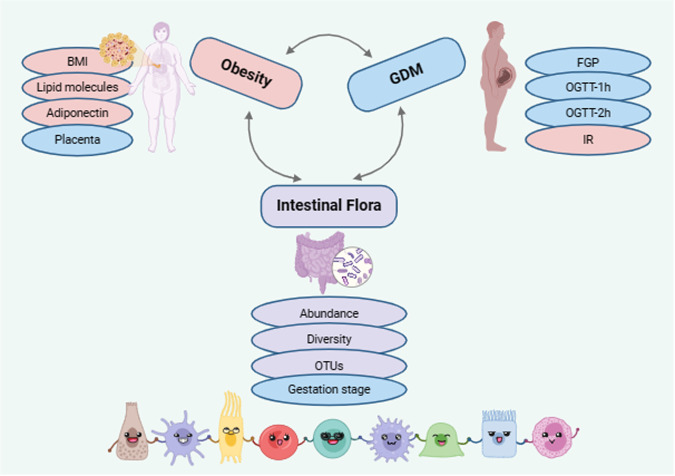
Association of intestinal microbiota and obesity with GDM.

This review presents an overview of the gastrointestinal microbiota and its connection to obesity and diabetes (refer to Fig. 3), with a causal relationship between these three metabolic conditions. Differences between countries, regions, and ethnicities were analyzed. Currently, no specific combination of gastrointestinal microbiota has an absolute advantage to the host. However, there is some predictive significance for the degree of obesity and the severity of GDM based on the up- or down-regulation, expression or non-expression, and changes in abundance and diversity of different gastrointestinal microbiomes. The placenta plays a pivotal role during this particular period of pregnancy. Additionally, research on BA and intestinal flora mechanisms is a current topic in the field of metabolic diseases. The detection of gastrointestinal microbiome is becoming increasingly important. It can be used as a clinical indicator to assist in the diagnosis of obstetrics and gynecology, especially in patients with GDM.

However, this study also has some limitations: The investigation did not cover whether the offspring of mothers with GDM have abnormal intestinal microbiome; Additionally, this review did not discuss the potential of probiotics to treat intestinal dysbiosis due to length constraints; Furthermore, the limited number of current literatures on the relationship between the three factors makes it impossible to carry out a meta-analysis. Considering the aforementioned limitations, it is expected that future studies will concentrate on this aspect to provide advantages to patients with GDM.

Throughout the extensive history of studying gastrointestinal microbiome, it has been discovered that certain combinations or individual microbes have significant effects on host metabolism. This study analyzed high-quality and related literatures to identify that an imbalance in the F/B ratio may be a characteristic feature of intestinal microbiome dysbiosis, as outlined below: 1. An imbalance of the F/B ratio may lead to metabolic disorders such as obesity and diabetes; 2. The F/B ratio has been found to decrease with age, which may result in decreased glucose tolerance; 3. It can be concluded that the alteration of the Firmicutes is a characteristic marker of both GDM and obesity. Additionally, a characteristic biomarker of GDM, Ruminococcus gnavus, was found. However, the up- or down-regulation of the Firmicutes did not consistently affect the development of the disease. Studies have shown conflicting results regarding the F/B ratio in obese and GDM patients, with some suggesting a decrease and others suggesting a trend towards an increase in overweight or obesity. Despite some results from animal studies contradicting the transplantation of microbiota in obese populations, further research has shown promise. With the deepening and refinement of animal studies, as well as large-scale population-based studies (which are mainly retrospective at present), it is believed that specific changes in human intestinal microbiome (especially in pregnant populations) will be explored. This will be a milestone for early identification of clinical diseases and effective monitoring of health status.

### Supplementary information

Abbreviations

## References

[1] Kolodziejczyk AA, Zheng D, Elinav E (2019). Diet-microbiota interactions and personalized nutrition. Nat Rev Microbiol.

[2] Qin Y, Roberts JD, Grimm SA, Lih FB, Deterding LJ, Li R (2018). An obesity-associated gut microbiome reprograms the intestinal epigenome and leads to altered colonic gene expression. Genome Biol.

[3] Gilbert JA, Blaser MJ, Caporaso JG, Jansson JK, Lynch SV, Knight R (2018). Current understanding of the human microbiome. Nat Med.

[4] Song Z, Li S, Li R (2022). An Investigation into the Correlation of Intestinal Flora with Obesity and Gestational Diabetes Mellitus. Comput Math Methods Med.

[5] Rinninella E, Raoul P, Cintoni M, Franceschi F, Miggiano GAD, Gasbarrini A (2019). What is the Healthy Gut Microbiota Composition? A Changing Ecosystem across Age, Environment, Diet, and Diseases. Microorganisms.

[6] Li D, Wang P, Wang P, Hu X, Chen F (2016). The gut microbiota: A treasure for human health. Biotechnol Adv.

[7] McIntyre HD, Catalano P, Zhang C, Desoye G, Mathiesen ER, Damm P (2019). Gestational diabetes mellitus. Nat Rev Dis Prim.

[8] Zheng QX, Wang HW, Jiang XM, Lin Y, Liu GH, Pan M (2022). Prepregnancy body mass index and gestational weight gain are associated with maternal and infant adverse outcomes in Chinese women with gestational diabetes. Sci Rep.

[9] Committee on Practice Bulletins—Obstetrics. ACOG Practice Bulletin No. 190: Gestational Diabetes Mellitus. Obstet Gynecol. 2018;131: e49–e64. 10.1097/AOG.0000000000002501 29370047

[10] Pervjakova N, Moen GH, Borges MC, Ferreira T, Cook JP, Allard C (2022). Multi-ancestry genome-wide association study of gestational diabetes mellitus highlights genetic links with type 2 diabetes. Hum Mol Genet.

[11] Saeedi P, Petersohn I, Salpea P, Malanda B, Karuranga S, Unwin N (2019). Global and regional diabetes prevalence estimates for 2019 and projections for 2030 and 2045: Results from the International Diabetes Federation Diabetes Atlas, 9th edition. Diabetes Res Clin Pr.

[12] Fakhrul-Alam M, Sharmin-Jahan, Mashfiqul-Hasan, Nusrat-Sultana, Mohona-Zaman, Rakibul-Hasan M (2020). Insulin secretory defect may be the major determinant of GDM in lean mothers. J Clin Transl Endocrinol.

[13] Lynch SV, Pedersen O (2016). The Human Intestinal Microbiome in Health and Disease. N Engl J Med.

[14] Abbasalizad Farhangi M, Vajdi M (2021). Gut microbiota-associated trimethylamine N-oxide and increased cardiometabolic risk in adults: a systematic review and dose-response meta-analysis. Nutr Rev.

[15] Ziętek M, Celewicz Z, Szczuko M (2021). Short-Chain Fatty Acids, Maternal Microbiota and Metabolism in Pregnancy. Nutrients.

[16] Frank DN, St Amand AL, Feldman RA, Boedeker EC, Harpaz N, Pace NR (2007). Molecular-phylogenetic characterization of microbial community imbalances in human inflammatory bowel diseases. Proc Natl Acad Sci USA.

[17] Thaiss CA, Itav S, Rothschild D, Meijer MT, Levy M, Moresi C (2016). Persistent microbiome alterations modulate the rate of post-dieting weight regain. Nature.

[18] Gomes AC, Hoffmann C, Mota JF (2018). The human gut microbiota: Metabolism and perspective in obesity. Gut Microbes.

[19] Crusell MKW, Hansen TH, Nielsen T, Allin KH, Rühlemann MC, Damm P (2018). Gestational diabetes is associated with change in the gut microbiota composition in third trimester of pregnancy and postpartum. Microbiome.

[20] Li R, Andreu-Sánchez S, Kuipers F, Fu J (2021). Gut microbiome and bile acids in obesity-related diseases. Best Pr Res Clin Endocrinol Metab.

[21] Fogelson KA, Dorrestein PC, Zarrinpar A, Knight R (2023). The Gut Microbial Bile Acid Modulation and Its Relevance to Digestive Health and Diseases. Gastroenterology.

[22] Matsubara T, Li F, Gonzalez FJ (2013). FXR signaling in the enterohepatic system. Mol Cell Endocrinol.

[23] Pols TW, Noriega LG, Nomura M, Auwerx J, Schoonjans K (2011). The bile acid membrane receptor TGR5 as an emerging target in metabolism and inflammation. J Hepatol.

[24] Watanabe M, Houten SM, Mataki C, Christoffolete MA, Kim BW, Sato H (2006). Bile acids induce energy expenditure by promoting intracellular thyroid hormone activation. Nature.

[25] Fiorucci S, Mencarelli A, Palladino G, Cipriani S (2009). Bile-acid-activated receptors: targeting TGR5 and farnesoid-X-receptor in lipid and glucose disorders. Trends Pharm Sci.

[26] Katsuma S, Hirasawa A, Tsujimoto G (2005). Bile acids promote glucagon-like peptide-1 secretion through TGR5 in a murine enteroendocrine cell line STC-1. Biochem Biophys Res Commun.

[27] Duran-Sandoval D, Cariou B, Percevault F, Hennuyer N, Grefhorst A, van Dijk TH (2005). The farnesoid X receptor modulates hepatic carbohydrate metabolism during the fasting-refeeding transition. J Biol Chem.

[28] Wahlström A, Sayin SI, Marschall HU, Bäckhed F (2016). Intestinal Crosstalk between Bile Acids and Microbiota and Its Impact on Host Metabolism. Cell Metab.

[29] Ma K, Saha PK, Chan L, Moore DD (2006). Farnesoid X receptor is essential for normal glucose homeostasis. J Clin Invest.

[30] Lambert G, Amar MJ, Guo G, Brewer HB, Gonzalez FJ, Sinal CJ (2003). The farnesoid X-receptor is an essential regulator of cholesterol homeostasis. J Biol Chem.

[31] Lee A, Ader M, Bray GA, Bergman RN (1992). Diurnal variation in glucose tolerance. Cyclic suppression of insulin action and insulin secretion in normal-weight, but not obese, subjects. Diabetes.

[32] Guo X, Wang J, Xu H, Wang Y, Cao Y, Wen Y (2024). Obesity induced disruption on diurnal rhythm of insulin sensitivity via gut microbiome-bile acid metabolism. Biochim Biophys Acta Mol Cell Biol Lipids.

[33] Tilg H, Adolph TE (2015). Influence of the human intestinal microbiome on obesity and metabolic dysfunction. Curr Opin Pediatr.

[34] Neuman H, Koren O (2017). The Pregnancy Microbiome. Nestle Nutr Inst Workshop Ser.

[35] Clemente JC, Pehrsson EC, Blaser MJ, Sandhu K, Gao Z, Wang B (2015). The microbiome of uncontacted Amerindians. Sci Adv.

[36] Su Y, Chen L, Zhang DY, Gan XP, Cao YN, Cheng DC (2021). The characteristics of intestinal flora in overweight pregnant women and the correlation with gestational diabetes mellitus. Endocr Connect.

[37] Moran-Ramos S, López-Contreras BE, Canizales-Quinteros S (2017). Gut Microbiota in Obesity and Metabolic Abnormalities: A Matter of Composition or Functionality?. Arch Med Res.

[38] Jovel J, Patterson J, Wang W, Hotte N, O’Keefe S, Mitchel T (2016). Characterization of the Gut Microbiome Using 16S or Shotgun Metagenomics. Front Microbiol.

[39] Mullins TP, Tomsett KI, Gallo LA, Callaway LK, McIntyre HD, Dekker Nitert M (2021). Maternal gut microbiota displays minor changes in overweight and obese women with GDM. Nutr Metab Cardiovasc Dis.

[40] Abdullah B, Daud S, Aazmi MS, Idorus MY, Mahamooth MIJ (2022). Gut microbiota in pregnant Malaysian women: a comparison between trimesters, body mass index and gestational diabetes status. BMC Pregnancy Childbirth.

[41] Vavreckova M, Galanova N, Kostovcik M, Krystynik O, Ivanovova E, Roubalova R (2022). Specific gut bacterial and fungal microbiota pattern in the first half of pregnancy is linked to the development of gestational diabetes mellitus in the cohort including obese women. Front Endocrinol.

[42] Dualib PM, Taddei CR, Fernandes G, Carvalho CRS, Sparvoli LG, Silva IT (2022). Gut Microbiota across Normal Gestation and Gestational Diabetes Mellitus: A Cohort Analysis. Metabolites.

[43] Metzger BE, Gabbe SG, Persson B, Buchanan TA, Catalano PA, International Association of Diabetes and Pregnancy Study Groups Consensus Panel (2010). International association of diabetes and pregnancy study groups recommendations on the diagnosis and classification of hyperglycemia in pregnancy. Diabetes Care.

[44] Le Chatelier E, Nielsen T, Qin J, Prifti E, Hildebrand F, Falony G (2013). Richness of human gut microbiome correlates with metabolic markers. Nature.

[45] Blaut M (2015). Gut microbiota and energy balance: role in obesity. Proc Nutr Soc.

[46] Ma Q, Li Y, Li P, Wang M, Wang J, Tang Z (2019). Research progress in the relationship between type 2 diabetes mellitus and intestinal flora. Biomed Pharmacother.

[47] Kimura I, Ozawa K, Inoue D, Imamura T, Kimura K, Maeda T (2013). The gut microbiota suppresses insulin-mediated fat accumulation via the short-chain fatty acid receptor GPR43. Nat Commun.

[48] Tolhurst G, Heffron H, Lam YS, Parker HE, Habib AM, Diakogiannaki E (2012). Short-chain fatty acids stimulate glucagon-like peptide-1 secretion via the G-protein-coupled receptor FFAR2. Diabetes.

[49] Ye G, Zhang L, Wang M, Chen Y, Gu S, Wang K (2019). The Gut Microbiota in Women Suffering from Gestational Diabetes Mellitus with the Failure of Glycemic Control by Lifestyle Modification. J Diabetes Res.

[50] Kuang YS, Lu JH, Li SH, Li JH, Yuan MY, He JR (2017). Connections between the human gut microbiome and gestational diabetes mellitus. Gigascience.

[51] Cortez RV, Taddei CR, Sparvoli LG, Ângelo AGS, Padilha M, Mattar R (2019). Microbiome and its relation to gestational diabetes. Endocrine..

[52] Gao B, Zhong M, Shen Q, Wu Y, Cao M, Ju S (2020). Gut microbiota in early pregnancy among women with Hyperglycaemia vs. Normal blood glucose. BMC Pregnancy Childbirth.

[53] Nuriel-Ohayon M, Neuman H, Koren O (2016). Microbial Changes during Pregnancy, Birth, and Infancy. Front Microbiol.

[54] Ferrocino I, Ponzo V, Gambino R, Zarovska A, Leone F, Monzeglio C (2018). Changes in the gut microbiota composition during pregnancy in patients with gestational diabetes mellitus (GDM). Sci Rep.

[55] Koren O, Goodrich JK, Cullender TC, Spor A, Laitinen K, Bäckhed HK (2012). Host remodeling of the gut microbiome and metabolic changes during pregnancy. Cell.

[56] Liu J, Yang H, Yin Z, Jiang X, Zhong H, Qiu D (2017). Remodeling of the gut microbiota and structural shifts in Preeclampsia patients in South China. Eur J Clin Microbiol Infect Dis.

[57] Li G, Yin P, Chu S, Gao W, Cui S, Guo S (2021). Correlation Analysis between GDM and Gut Microbial Composition in Late Pregnancy. J Diabetes Res.

[58] Wang X, Liu H, Li Y, Huang S, Zhang L, Cao C (2020). Altered gut bacterial and metabolic signatures and their interaction in gestational diabetes mellitus. Gut Microbes.

[59] Qin J, Li Y, Cai Z, Li S, Zhu J, Zhang F (2012). A metagenome-wide association study of gut microbiota in type 2 diabetes. Nature.

[60] Huang X, Fang S, Yang H, Gao J, He M, Ke S (2017). Evaluating the contribution of gut microbiome to the variance of porcine serum glucose and lipid concentration. Sci Rep.

[61] Juge N (2023). Microbe Profile: Ruminococcus gnavus: the yin and yang of human gut symbionts. Microbiology.

[62] Ponzo V, Fedele D, Goitre I, Leone F, Lezo A, Monzeglio C (2019). Diet-Gut Microbiota Interactions and Gestational Diabetes Mellitus (GDM). Nutrients.

[63] Huang L, Sililas P, Thonusin C, Tongsong T, Luewan S, Chattipakorn N (2022). Association Between Gut Microbiota and Insulin Therapy in Women With Gestational Diabetes Mellitus. Can J Diabetes.

[64] Li H, Shi J, Zhao L, Guan J, Liu F, Huo G (2021). Lactobacillus plantarum KLDS1.0344 and Lactobacillus acidophilus KLDS1.0901 Mixture Prevents Chronic Alcoholic Liver Injury in Mice by Protecting the Intestinal Barrier and Regulating Gut Microbiota and Liver-Related Pathways. J Agric Food Chem.

[65] Liu J, Li T, Wu H, Shi H, Bai J, Zhao W (2019). Lactobacillus rhamnosus GG strain mitigated the development of obstructive sleep apnea-induced hypertension in a high salt diet via regulating TMAO level and CD4+ T cell induced-type I inflammation. Biomed Pharmacother.

[66] Jang HM, Han SK, Kim JK, Oh SJ, Jang HB, Kim DH (2019). Lactobacillus sakei Alleviates High-Fat-Diet-Induced Obesity and Anxiety in Mice by Inducing AMPK Activation and SIRT1 Expression and Inhibiting Gut Microbiota-Mediated NF-κB Activation. Mol Nutr Food Res.

[67] Wu Y, Bible PW, Long S, Ming WK, Ding W, Long Y (2020). Metagenomic analysis reveals gestational diabetes mellitus-related microbial regulators of glucose tolerance. Acta Diabetol.

[68] Chen F, Gan Y, Li Y, He W, Wu W, Wang K (2021). Association of gestational diabetes mellitus with changes in gut microbiota composition at the species level. BMC Microbiol.

[69] Nehra V, Allen JM, Mailing LJ, Kashyap PC, Woods JA (2016). Gut Microbiota: Modulation of Host Physiology in Obesity. Physiology.

[70] Carlos D, Pérez MM, Leite JA, Rocha FA, Martins LMS, Pereira CA (2020). NOD2 Deficiency Promotes Intestinal CD4+ T Lymphocyte Imbalance, Metainflammation, and Aggravates Type 2 Diabetes in Murine Model. Front Immunol.

[71] Garcia-Mantrana I, Collado MC (2016). Obesity and overweight: Impact on maternal and milk microbiome and their role for infant health and nutrition. Mol Nutr Food Res.

[72] Spor A, Koren O, Ley R (2011). Unravelling the effects of the environment and host genotype on the gut microbiome. Nat Rev Microbiol.

[73] Bäckhed F, Ding H, Wang T, Hooper LV, Koh GY, Nagy A (2004). The gut microbiota as an environmental factor that regulates fat storage. Proc Natl Acad Sci USA.

[74] Arumugam M, Raes J, Pelletier E, Le Paslier D, Yamada T, Mende DR (2011). Enterotypes of the human gut microbiome. Nature.

[75] Sehgal K, Khanna S (2021). Gut microbiota: a target for intervention in obesity. Expert Rev Gastroenterol Hepatol.

[76] Edwards SM, Cunningham SA, Dunlop AL, Corwin EJ (2017). The Maternal Gut Microbiome During Pregnancy. MCN Am J Matern Child Nurs.

[77] Riva A, Borgo F, Lassandro C, Verduci E, Morace G, Borghi E (2017). Pediatric obesity is associated with an altered gut microbiota and discordant shifts in Firmicutes populations. Environ Microbiol.

[78] Ley RE, Bäckhed F, Turnbaugh P, Lozupone CA, Knight RD, Gordon JI (2005). Obesity alters gut microbial ecology. Proc Natl Acad Sci USA.

[79] Kasai C, Sugimoto K, Moritani I, Tanaka J, Oya Y, Inoue H (2015). Comparison of the gut microbiota composition between obese and non-obese individuals in a Japanese population, as analyzed by terminal restriction fragment length polymorphism and next-generation sequencing. BMC Gastroenterol.

[80] Armougom F, Henry M, Vialettes B, Raccah D, Raoult D (2009). Monitoring bacterial community of human gut microbiota reveals an increase in Lactobacillus in obese patients and Methanogens in anorexic patients. PLoS One.

[81] Million M, Thuny F, Angelakis E, Casalta JP, Giorgi R, Habib G (2013). Lactobacillus reuteri and Escherichia coli in the human gut microbiota may predict weight gain associated with vancomycin treatment. Nutr Diabetes.

[82] Vallianou N, Stratigou T, Christodoulatos GS, Dalamaga M (2019). Understanding the Role of the Gut Microbiome and Microbial Metabolites in Obesity and Obesity-Associated Metabolic Disorders: Current Evidence and Perspectives. Curr Obes Rep.

[83] Ley RE, Turnbaugh PJ, Klein S, Gordon JI (2006). Microbial ecology: human gut microbes associated with obesity. Nature.

[84] Turnbaugh PJ, Ley RE, Mahowald MA, Magrini V, Mardis ER, Gordon JI (2006). An obesity-associated gut microbiome with increased capacity for energy harvest. Nature.

[85] Zacarías MF, Collado MC, Gómez-Gallego C, Flinck H, Aittoniemi J, Isolauri E (2018). Pregestational overweight and obesity are associated with differences in gut microbiota composition and systemic inflammation in the third trimester. PLoS One.

[86] Houttu N, Mokkala K, Laitinen K (2018). Overweight and obesity status in pregnant women are related to intestinal microbiota and serum metabolic and inflammatory profiles. Clin Nutr.

[87] Rodrigues VF, Elias-Oliveira J, Pereira ÍS, Pereira JA, Barbosa SC, Machado MSG (2022). Akkermansia muciniphila and Gut Immune System: A Good Friendship That Attenuates Inflammatory Bowel Disease, Obesity, and Diabetes. Front Immunol.

[88] Lim MY, You HJ, Yoon HS, Kwon B, Lee JY, Lee S (2017). The effect of heritability and host genetics on the gut microbiota and metabolic syndrome. Gut.

[89] Dao MC, Everard A, Aron-Wisnewsky J, Sokolovska N, Prifti E, Verger EO (2016). Akkermansia muciniphila and improved metabolic health during a dietary intervention in obesity: relationship with gut microbiome richness and ecology. Gut.

[90] Depommier C, Van Hul M, Everard A, Delzenne NM, De Vos WM, Cani PD (2020). Pasteurized Akkermansia muciniphila increases whole-body energy expenditure and fecal energy excretion in diet-induced obese mice. Gut Microbes.

[91] Yoon HS, Cho CH, Yun MS, Jang SJ, You HJ, Kim JH (2021). Akkermansia muciniphila secretes a glucagon-like peptide-1-inducing protein that improves glucose homeostasis and ameliorates metabolic disease in mice. Nat Microbiol.

[92] Lee JS, Song WS, Lim JW, Choi TR, Jo SH, Jeon HJ (2022). An integrative multiomics approach to characterize anti-adipogenic and anti-lipogenic effects of Akkermansia muciniphila in adipocytes. Biotechnol J.

[93] Plows JF, Stanley JL, Baker PN, Reynolds CM, Vickers MH (2018). The Pathophysiology of Gestational Diabetes Mellitus. Int J Mol Sci.

[94] Pantham P, Aye IL, Powell TL (2015). Inflammation in maternal obesity and gestational diabetes mellitus. Placenta.

[95] Parrettini S, Caroli A, Torlone E (2020). Nutrition and Metabolic Adaptations in Physiological and Complicated Pregnancy: Focus on Obesity and Gestational Diabetes. Front Endocrinol.

[96] Gregor MF, Hotamisligil GS (2011). Inflammatory mechanisms in obesity. Annu Rev Immunol.

[97] Wang Y, Pan XF, Pan A (2023). Lipidomics in gestational diabetes mellitus. Curr Opin Lipido.

[98] Wang Y, Wu P, Huang Y, Ye Y, Yang X, Sun F (2022). BMI and lipidomic biomarkers with risk of gestational diabetes in pregnant women. Obesity.

[99] MacDonald SC, Bodnar LM, Himes KP, Hutcheon JA (2017). Patterns of Gestational Weight Gain in Early Pregnancy and Risk of Gestational Diabetes Mellitus. Epidemiology.

[100] Jarvie E, Hauguel-de-Mouzon S, Nelson SM, Sattar N, Catalano PM, Freeman DJ (2010). Lipotoxicity in obese pregnancy and its potential role in adverse pregnancy outcome and obesity in the offspring. Clin Sci.

[101] Kampmann U, Knorr S, Fuglsang J, Ovesen P (2019). Determinants of Maternal Insulin Resistance during Pregnancy: An Updated Overview. J Diabetes Res.

[102] Chatuphonprasert W, Jarukamjorn K, Ellinger I (2018). Physiology and Pathophysiology of Steroid Biosynthesis, Transport and Metabolism in the Human Placenta. Front Pharm.

[103] Dubé E, Ethier-Chiasson M, Lafond J (2013). Modulation of cholesterol transport by insulin-treated gestational diabetes mellitus in human full-term placenta. Biol Reprod.

[104] Bouvier D, Rouzaire M, Marceau G, Prat C, Pereira B, Lemarié R (2015). Aquaporins and Fetal Membranes From Diabetic Parturient Women: Expression Abnormalities and Regulation by Insulin. J Clin Endocrinol Metab.

[105] Zhang Y, Ye J, Fan J (2017). Regulation of malonyl-CoA-acyl carrier protein transacylase network in umbilical cord blood affected by intrauterine hyperglycemia. Oncotarget..

[106] Tanaka K, Yamada K, Matsushima M, Izawa T, Furukawa S, Kobayashi Y (2018). Increased maternal insulin resistance promotes placental growth and decreases placental efficiency in pregnancies with obesity and gestational diabetes mellitus. J Obstet Gynaecol Res.

[107] Huang L, Liu J, Feng L, Chen Y, Zhang J, Wang W (2014). Maternal prepregnancy obesity is associated with higher risk of placental pathological lesions. Placenta..

[108] Huynh J, Dawson D, Roberts D, Bentley-Lewis R (2015). A systematic review of placental pathology in maternal diabetes mellitus. Placenta..

[109] Salomon C, Scholz-Romero K, Sarker S, Sweeney E, Kobayashi M, Correa P (2016). Gestational Diabetes Mellitus Is Associated With Changes in the Concentration and Bioactivity of Placenta-Derived Exosomes in Maternal Circulation Across Gestation. Diabetes..

[110] Rice GE, Scholz-Romero K, Sweeney E, Peiris H, Kobayashi M, Duncombe G (2015). The Effect of Glucose on the Release and Bioactivity of Exosomes From First Trimester Trophoblast Cells. J Clin Endocrinol Metab.

[111] Greenhill C (2017). Diabetes: The role of adiponectin in gestational diabetes mellitus. Nat Rev Endocrinol.

[112] Miehle K, Stepan H, Fasshauer M (2012). Leptin, adiponectin and other adipokines in gestational diabetes mellitus and pre-eclampsia. Clin Endocrinol.

[113] Haghiac M, Basu S, Presley L, Serre D, Catalano PM, Hauguel-de Mouzon S (2014). Patterns of adiponectin expression in term pregnancy: impact of obesity. J Clin Endocrinol Metab.

[114] Qiao L, Wattez JS, Lee S, Nguyen A, Schaack J, Hay WW (2017). Adiponectin Deficiency Impairs Maternal Metabolic Adaptation to Pregnancy in Mice. Diabetes..

[115] Turer AT, Scherer PE (2012). Adiponectin: mechanistic insights and clinical implications. Diabetologia..

[116] Bhograj A, Suryanarayana KM, Nayak A, Murthy NS, Dharmalingam M, Kalra P (2016). Serum adiponectin levels in gestational diabetes mellitus. Indian J Endocrinol Metab.

[117] Mohammadi T, Paknahad Z (2017). Adiponectin Concentration in Gestational Diabetic Women: a Case-Control Study. Clin Nutr Res.

