# Supplementary material for: Association of gastrointestinal microbiome and obesity with gestational diabetes mellitus-an updated globally based review of the high-quality literatures
Source: Nutr Diabetes. 2024 May 21;14:31. doi: 10.1038/s41387-024-00291-5 (PMC11109140; doi:10.1038/s41387-024-00291-5)
Supplement: Supplementary file 1 — Abbreviations [file 41387_2024_291_MOESM1_ESM.docx]

**Abbreviations**

| GDM | gestational diabetes mellitus |
| --- | --- |
| BMI | body mass index |
| IR | insulin resistance |
| T3 | the third trimester of pregnancy |
| BA | bile acids |
| FXR | farnesoid X receptor |
| TGR5 | G protein-coupled BA receptor 1 |
| GLP-1 | glucagon-like peptide-1 |
| F/B | The ratio of Firmicutes to Bacteroides |
| OGTT_1h | glucose levels at 1 hour |
| OGTT_2h | glucose levels at 2 hours |
| SCFAs | Short-chain fatty acids |
| GPCRs | G protein-coupled receptors |
| T1 | the first trimester of pregnancy |
| T2 | mid-pregnancy |
| OTU | operational taxonomic unit |
